# Cell Systems Bioelectricity: How Different Intercellular Gap Junctions Could Regionalize a Multicellular Aggregate

**DOI:** 10.3390/cancers13215300

**Published:** 2021-10-22

**Authors:** Alejandro Riol, Javier Cervera, Michael Levin, Salvador Mafe

**Affiliations:** 1 Dept. Termodinàmica, Facultat de Física, Universitat de València, E-46100 Burjassot, Spain; alejandro.riol@uv.es; 2 Dept. of Biology, Allen Discovery Center at Tufts University, Medford, MA 02155-4243, USA; Michael.Levin@tufts.edu

**Keywords:** cell bioelectricity, electric potential patterns, ion channels, intercellular gap junctions, tumorigenesis

## Abstract

**Simple Summary:**

Electric potential patterns across tissues are instructive for development, regeneration, and tumorigenesis because they can influence transcription, migration, and differentiation through biochemical and biomechanical downstream processes. Determining the origins of the spatial domains of distinct potential, which in turn decide anatomical features such as limbs, eyes, brain, and heart, is critical to a mature understanding of how bioelectric signaling drives morphogenesis. We studied theoretically how connexin proteins with different voltage-gated gap junction conductances can maintain multicellular regions at distinct membrane potentials. We analyzed a minimal model that incorporates effective conductances ultimately related to specific ion channel and junction proteins that are amenable to external regulation. We also consider a bioelectrical relationship between the connexin composition of the intercellular gap junction and different stages of cancer.

**Abstract:**

Electric potential distributions can act as instructive pre-patterns for development, regeneration, and tumorigenesis in cell systems. The biophysical states influence transcription, proliferation, cell shape, migration, and differentiation through biochemical and biomechanical downstream transduction processes. A major knowledge gap is the origin of spatial patterns in vivo, and their relationship to the ion channels and the electrical synapses known as gap junctions. Understanding this is critical for basic evolutionary developmental biology as well as for regenerative medicine. We computationally show that cells may express connexin proteins with different voltage-gated gap junction conductances as a way to maintain multicellular regions at distinct membrane potentials. We show that increasing the multicellular connectivity via enhanced junction function does not always contribute to the bioelectrical normalization of abnormally depolarized multicellular patches. From a purely electrical junction view, this result suggests that the reduction rather than the increase of specific connexin levels can also be a suitable bioelectrical approach in some cases and time stages. We offer a minimum model that incorporates effective conductances ultimately related to specific ion channel and junction proteins that are amenable to external regulation. We suggest that the bioelectrical patterns and their encoded instructive information can be externally modulated by acting on the mean fields of cell systems, a complementary approach to that of acting on the molecular characteristics of individual cells. We believe that despite the limitations of a biophysically focused model, our approach can offer useful qualitative insights into the collective dynamics of cell system bioelectricity.

## 1. Introduction

Patterns in biology result from the interplay between biochemical [1] and biomechanical [2] signals that establish spatio-temporal correlations in multicellular aggregates. In addition, a complementary distributed control based on the cell membrane bioelectricity is also emerging [3,4,5,6,7]. The cell potential *V*, defined as the electric potential difference between the cell inside and the external microenvironment, regulates the distribution of signaling cations and biochemical messengers. In turn, *V* is also modulated by these distributions because of their influence on the transcription of ion channel proteins, which constitutes a bioelectrical feedback control at the single-cell scale [3,4,5,8].

At the multicellular scale, two general mechanisms for cells coordination are the extracellular signaling and the intercellular gap junctions. The connexin proteins forming the gap junction channels allow the latter communication by the exchange of biochemical and electrical signals between adjacent cells [5,7,9,10,11,12,13,14]. Different junction types can be identified on the basis of their constituent proteins and distinct voltage-gated conductances. In the case of bioelectrical coupling, it has been shown that multicellular mean fields can collectively influence downstream transcriptional processes via the spatio-temporal maps of biochemical messengers which are dictated by the local electric potentials [3,4,5,7,15]. Bioelectric signaling has now been shown to be instructive for morphogenesis: specific changes in membrane potential patterns can induce new organ formation such as eye, trigger the regeneration of whole appendages, repair the brain after mutation or the exposure to teratogens, and normalize tumors [16,17,18]. While much effort has gone into identifying the transduction machinery by which voltage changes influence second messenger pathways [9], it is much less clear what factors control the shape, size, and location of specific bioelectric domains that serve as pre-patterns laying out the face, brain, and other organs [19,20]. 

We aim here at theoretically exploring how the spatial distribution of different gap junctions can maintain regions of cells in distinct bioelectrical states, thus regionalizing tissue into specific patterns. We consider different scenarios that concern the single-cell bioelectrical characteristics, the different gating and coupling degree of the junctions, and the relative sizes of the regions forming the multicellular aggregate. The experimental motivation for the simulations is that electric potentials can act as instructive pre-patterns for morphogenesis [21,22,23] and tumor initiation and normalization [14,24,25], as suggested by Levin and others. Our bioelectrical approach can also be applied to interconnected smooth muscle cells that propagate signals over relatively long distances [26]. In addition, it permits both stationary and oscillating patterns [5,27], thus providing useful insights into a variety of multicellular systems including syncytia of astrocytes [4,28].

Note that some tumor and anti-tumor approaches based on connexin targeting are closely related to the *bystander community* effect that results from the gap junction intercellular coupling [14,29,30,31,32,33,34,35]. In particular, the differential expression of connexin subunits Cx46 and Cx43 between the cancer stem cells (CSCs) that contribute to tumor propagation and non-CSCs has been connected to distinct membrane potentials and cell differentiation processes [14]. The present simulations constitute a significant extension of those of reference [36] to the case of cell systems with different connexin proteins and gap junctions. We consider only the junctional role of connexin proteins as transducers of electrical signals, disregarding additional biochemical and biomechanical effects that can be relevant in other contexts.

## 2. Biophysical Model

The single-cell model has been experimentally motivated and described with detail previously [5,36]. It considers two generic populations of voltage-gated ion channels of maximum conductances $G_{\mathrm{pol}}^{o}$ and $G_{\mathrm{dep}}^{o}$ that act to establish the polarized and depolarized single-cell states. While a wide variety of channels can exist in a cell [37], voltage-gated channels are crucial to bioelectricity because they allow the counteracting dynamics typical of many physiological functions including pacemaking, neural slow-wave oscillations, circadian clocks, and bioelectrical oscillatory phenomena in artificial tissues [5,11,12,37,38]. Here, we introduce two phenomenological equations that can qualitatively describe the experimental *I*–*V* curves characteristic of voltage-gated channels [37,39]:

(1) $$I_{\mathrm{dep}}=G_{\mathrm{dep}}(V-E_{\mathrm{dep}})=\frac{G_{\mathrm{dep}}^{o}(V-E_{\mathrm{dep}})}{1+\exp[-z(V-V_{\mathrm{th}})/V_{T}]}$$

(2) $$I_{\mathrm{pol}}=G_{\mathrm{pol}}(V-E_{\mathrm{pol}})=\frac{G_{\mathrm{pol}}^{o}(V-E_{\mathrm{pol}})}{1+\exp[z(V-V_{\mathrm{th}})/V_{T}]}$$

Note that, in Equations (1) and (2), we have made explicit the dependence of the voltage-gated conductances $G_{\mathrm{dep}}$ and $G_{\mathrm{pol}}$ on the cell potential *V* [39]. Typical order of magnitude values are $G_{\mathrm{dep}}^{o}/G_{\mathrm{pol}}^{o}=1$ for the ratio of conductances, *z* = 3 for the effective gating charge of the channel, and $V_{\mathrm{th}}=-V_{T}=-(RT/F)=-27_{}^{}\;\mathrm{mV}$ for the threshold potentials, with *R* the gas constant, *T* the temperature, and *F* the Faraday constant [37,39]. The values introduced here for the cell polarized (*pol*) and depolarized (*dep*) equilibrium potentials are $E_{\mathrm{pol}}=-55_{}^{}\;\mathrm{mV}$ and $E_{\mathrm{dep}}=-5_{}^{}\;\mathrm{mV}$ [39]. Equations (1) and (2) permit to establish a bi-stable bioelectrical regime for an isolated cell, as previously described [36,39]. Note that, as shown in Figure 3a of [9], terminally differentiated somatic cells such as muscle cells tend to be polarized—they show negative membrane potentials—while embryonic, stem, and tumor cells tend to be depolarized. Thus, independently of the particular ionic species, gradients, and transporters that are involved in each case, we considered that the above potentials are representative of the *pol* and *dep* states, respectively [4,5].

Multicellular states depend not only on the single-cell channel conductances ratio $G_{\mathrm{dep}}^{o}/G_{\mathrm{pol}}^{o}$ and concentration-dependent potentials $E_{\mathrm{pol}}$ and $E_{\mathrm{dep}}$ but also on the intercellular gap junctions [5,39]. The bioelectrical states of neighboring cells are coupled by these conductances whose local states define the dynamic connectivity characteristic of multicellular patterns [5,39]. We aim here at extending our previous theoretical approach [36] to the biologically relevant case [14,26,40,41] of two different junction types that can be experimentally identified on the basis of their constituent family proteins and conductances. Note that vertebrate gap junctions are usually voltage-gated and thus their conductances depend on the intercellular potential difference rather than on the absolute potentials of the adjacent cells. In particular, high conductances are obtained when the two neighboring cells have similar cell potentials while low conductances are found when these cells are at different potentials [4,5,40,41].

**Figure 1:**
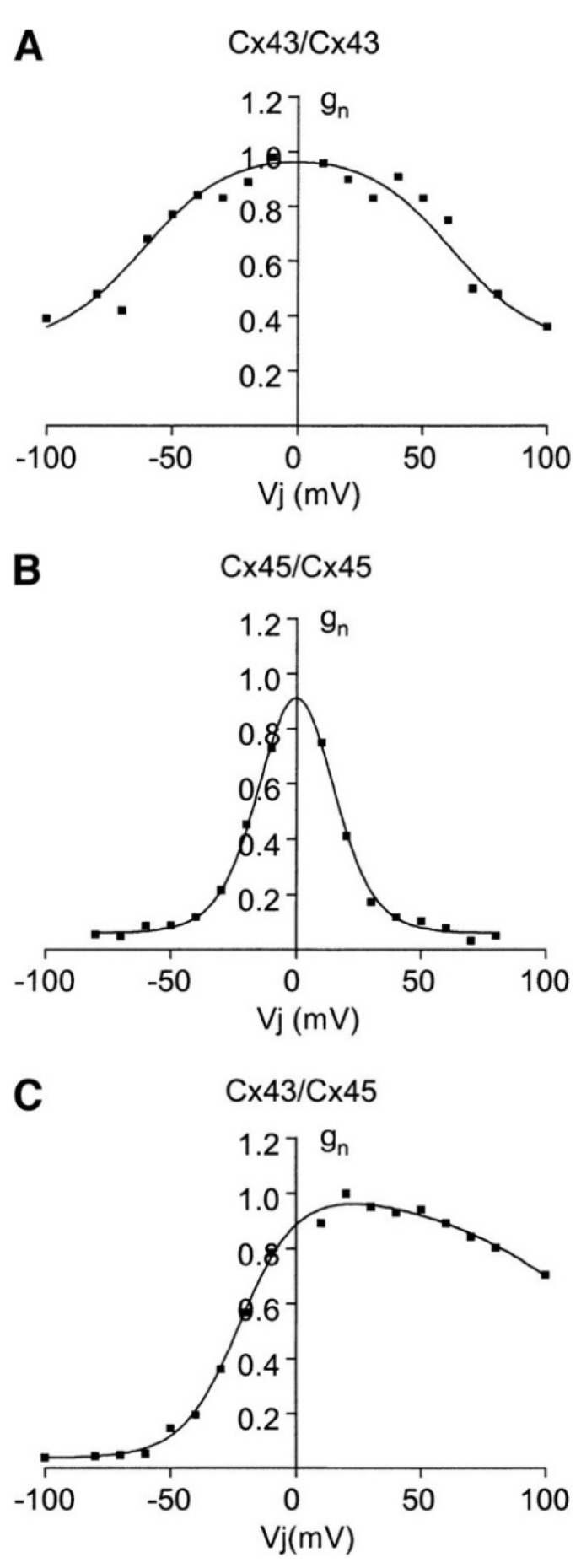
Normalized experimental junction conductances (g_n_) vs. voltage (Vj) of the homotypic Cx43/Cx43 (**A**) and Cx45/Cx45 (**B**) channels and the heterotypic Cx43/Cx45 (**C**) channel. Homotypic channels show symmetric conductances with different voltage-gated curve sharpness while the heterotypic Cx43/Cx45 channel shows an asymmetric conductance with an off-center peak. Reproduced with permission from [40]: Chen-Izu, Y.; Moreno, A.P.; Spangler, R.A. Opposing gates model for voltage gating of gap junction channels. *Am. J. Physiol. Cell Physiol.* **2001**, *281*, C1604–C1613.

**Figure 2:**
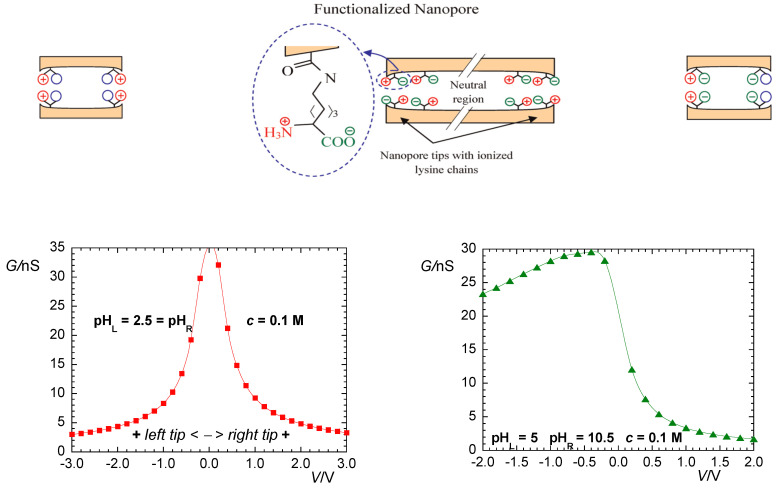
Sketch of the nanopore (not to scale) functionalized with amino acid lysine (*top*). Due to the geometry of the pore, the carboxylic acid and amino groups are concentrated close to the pore tips. Note that these pH-dependent charge groups are also present in connexin proteins. Conductance vs. voltage curves at 0.1 M KCl concentration with pH_L_ = pH_R_ = 2.5 (*bottom*, *left*) and pH_L_ = 5 and pH_R_ = 10.5 (*bottom*, *right*). The cartoons above each curve illustrate the distribution of fixed charges at the pore mouths (*top*). Experimental data taken from [43]: M. Ali, P. Ramirez, H Q. Nguyen, S. Nasir, J. Cervera, S. Mafe, W. Ensinger. Single cigar-shaped nanopores functionalized with amphoteric amino acid chains: experimental and theoretical characterization. *ACS Nano* **2012**, *6*, 3631–3640. Copyright 2012 American Chemical Society.

We will not address here the complex route between connexins, connexons, and gap junctions. Instead, we concentrate on the bioelectrical characteristics of voltage-gated gap junctions. Figure 1 shows the typical junction conductances observed for two homotypic Cx43/Cx43 and Cx45/Cx45 channels together with the case of the heterotypic Cx43/Cx45 channel [41]. Conductance changes in ion channels and junctions can be ascribed to voltage-gated structural rearrangements of the protein subunits along the pore. However, pH and ionic concentration effects are also important because they can modify the state of the pore charge moieties [37], as shown in Figure 2 for the case of a biomimetic nanopore [42,43]. Note that these pH-dependent charge groups are also present in connexin proteins.

Artificial biomimetic nanopores functionalized with amino acid chains show a qualitatively similar behavior, not only because of symmetric and asymmetric cross-sectional areas [42] but also because of the different charge distributions that can be externally established at the pore mouths [43]. Figure 2 shows the case of a symmetric cigar-shaped nanopore where, due to the particular pore geometry, the carboxylic acid and amino groups of lysine are concentrated close to the pore tips. In this case, the conductance vs. voltage curves measured at 0.1 M KCl salt concentration can be made symmetrical or asymmetrical according to the pH values of the left (L) and right (R) solutions.

The conductance gating of Figure 1 can be qualitatively reproduced by the phenomenological equation:

(3) $$G_{ij}=G_{1}+\frac{G_{2}}{\left\{1+\exp[-(V_{j}-V_{i}-V_{\mathrm{th},i})/V_{0,}{}_{i}]\right\}\left\{1+\exp[(V_{j}-V_{i}-V_{\mathrm{th},i})/V_{0,}{}_{j}]\right\}}$$

where $\left(V_{j}-V_{i}\right)$ is the intercellular electric potential difference between two adjacent cells *j* and *i* of potentials $V_{j}$ and $V_{i}$, $V_{\mathrm{th},j}$ and $V_{\mathrm{th},i}$ are the respective protein subunits threshold potentials, and the reference potentials $V_{0,j}$ and $V_{0,i}$ account for the different symmetry and sharpness of $G_{ij}$ observed in Figure 1. The conductances $G_{1}$ and $G_{2}$ are related to the minimum and maximum values of $G_{ij}$. The conductance of Equation (3) allows to modulate the multicellular network of coupled cells at both the single-cell transcriptional level and the intercellular post-translational level, as explained previously [5,36]. 

**Figure 3:**
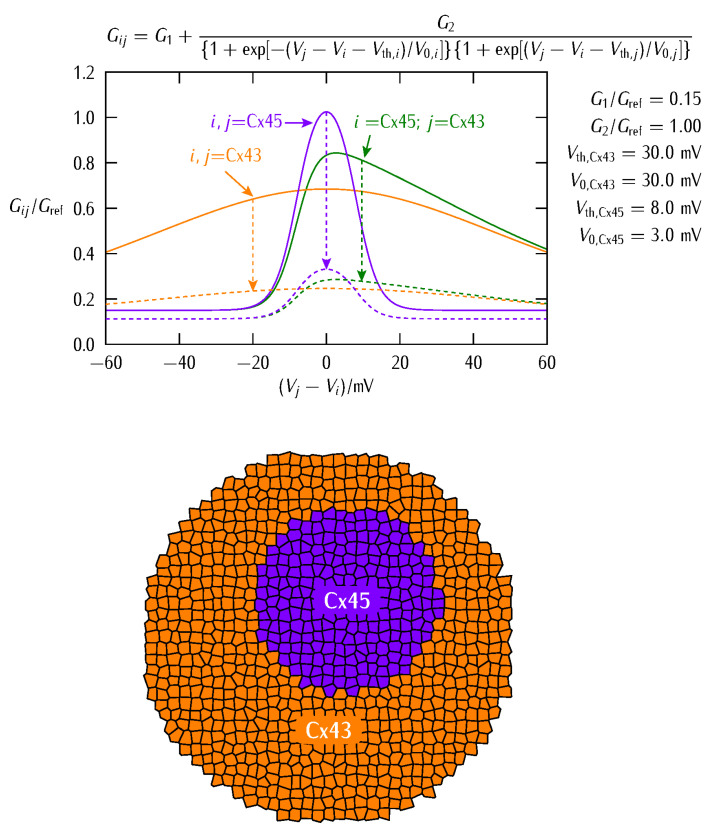
The gap junction conductances obtained from Equation (3) with the parameters shown in the figure (*top*). The symmetrical and asymmetrical cases of Figure 1 are considered together with the effect of decreasing the conductances $G_{1}$ and $G_{2}$ to the values $G_{1}^{’}=0.75G_{1}$ and $G_{2}^{’}=0.25G_{2}$ (*dashed lines*). This decrease may be due to an inhibition of the connexin expressions or the post-translational blocking of the junctions. The multicellular scheme shows the inner patch of *n* = 221 cells where connexin Cx45 is predominantly expressed surrounded by a bulk of *N* − *n* = 661 cells where connexin Cx43 is dominant (*bottom*), with *N* = 882 cells. Note that at the interface between the two cell regions with different homotypic gap junctions a heterotypic Cx43|Cx45 junction is formed.

Figure 3 (*top*) shows that Equation (3) qualitatively reproduces the experimental symmetrical and asymmetrical shapes of the different gap junctions $G_{ij}$ of Figure 1 [40,41]. Note that the values of $G_{ij}$ are scaled to a reference junction conductance $G_{\mathrm{ref}}$ to better show the model trends. The effect of decreasing the conductance parameters $G_{1}$ and $G_{2}$ to the values $G_{1}^{’}=0.75G_{1}$ and $G_{2}^{’}=0.25G_{2}$ is also shown (*dashed lines*). 

Figure 3 (*bottom*) schematically shows the two cell regions with the distinct families of connexin proteins used in the simulations. We assume that these regions are initially in different single-cell polarization states and study if the inner patch of abnormally depolarized cells (low absolute value of *V*) can resist normalization against the surrounding bulk of normally polarized cells (high absolute value of *V*). Note that all cells in the two regions share the same single-cell bioelectrical parameters, the only differences being their initially distinct polarized or depolarized stable states [5,39] and the region-dependent connexins expressions (Figure 3). 

The intercellular currents through the gap junction conductances of Equation (3) and Figure 3, together with the single-cell channel currents $I_{\mathrm{pol}}$ and $I_{\mathrm{dep}}$ of Equations (1) and (2), establish the individual cell potentials $V_{i}$(*i* = 1, 2, …, *N*) that change with time *t* according to the equation [5]:

(4) $$C_{i}\frac{dV_{i}}{dt}=-I_{\mathrm{pol}}-I_{\mathrm{dep}}+\sum_{\begin{array}{l} j_{}^{}\;\in \mathrm{nearest}\\ \mathrm{neighbors} \end{array}}G_{ij}(V_{j}-V_{i})$$

We restrict the sum to the nearest neighbors, assume an average number of one effective junction between two nearest neighbor cells, and introduce the single-cell capacitance $C_{i}=100_{}^{}\;\mathrm{pF}$. For a reference conductance $G_{\mathrm{ref}}^{o}=1_{}^{}\;\mathrm{nS}$, the single-cell electrical time is $\tau =C_{i}/G_{\mathrm{ref}}^{o}=0.1_{}^{}\;s$ only but the cell systems time can increase to 10–100 s for an aggregate composed of hundreds of cells [4,5].

Equation (4) gives multicellular mean fields that attempt to force the same polarization state for all cells in a highly connected region [4,5]. Experimentally, this result permits to encode instructive bioelectrical information over spatial regions of cells on the basis of their particular polarization states [3,4,5,9,15]. While the non-excitable cell dynamics of Equation (4) is slow compared with action potentials in neurons, the concepts of polarized and depolarized cell states, network connectivity, and multicellular patterns are common to both cell systems despite the differences in the information processing times. 

We will address here quasi-stationary patterns only, but the case of oscillatory regions modulated by the intercellular connectivity is described elsewhere [27]. Additionally, we have not considered the transcriptional regulation of the channel conductances because we studied it with detail previously [5,44]. This downstream regulation gives comparatively slow responses of the order of hours when transcriptional [44] and diffusional [39] processes are incorporated in the simulations. Experimentally, bioelectrical pre-patterns constitute templates for the spatio-temporal distributions of signaling ions and molecules that regulate the subsequent downstream biochemical processes [3,4,7]. 

## 3. Results and Discussion

We aim at providing qualitative insights into the fate of an abnormally depolarized inner patch initiated within a normally polarized multicellular aggregate when the intercellular coupling is changed. To this end, we describe the bioelectrical patterns that result from different decreases of the junction conductance (Figure 3, *top*). We might anticipate that weakening the intercellular coupling should favor the electrical regionalization initially established in Figure 3 (*bottom*) because of the decrease in the *interfacial* connectivity between the two regions. However, weakening the intercellular coupling also causes a decrease in the *internal* connectivity of each region and thus a decrease in the community effect that keeps the patch in the depolarized state. Thus, a delicate balance between these two opposing trends will eventually dictate the cell system fate. In addition to small cell systems, the modification of the intercellular coupling can also suggest mechanisms for establishing and modifying target patterns in tissue engineering [12,38]. 

Reprogramming of single-cell bioelectrical states may benefit from the mean field effects allowed by the intercellular coupling of multicellular aggregates [3,4,5,9]. Experimentally, adjacent domains of cells in different polarization states are characteristic of developmental and tumorigenic processes [3,4,9,45,46]. They can be established by local changes in gene expression [45] and spatial heterogeneities in the signaling molecules concentrations [46]. In our case, we consider an inner patch of initially depolarized cells surrounded by a bulk of polarized cells (Figure 3, *bottom*) at time *t* = 0. Subsequently, we simulate the multicellular aggregate changes that result from different junction conductance decreases affecting the system connectivity. To this end, we use Equations (1)–(4) and Figure 3. 

**Figure 4:**
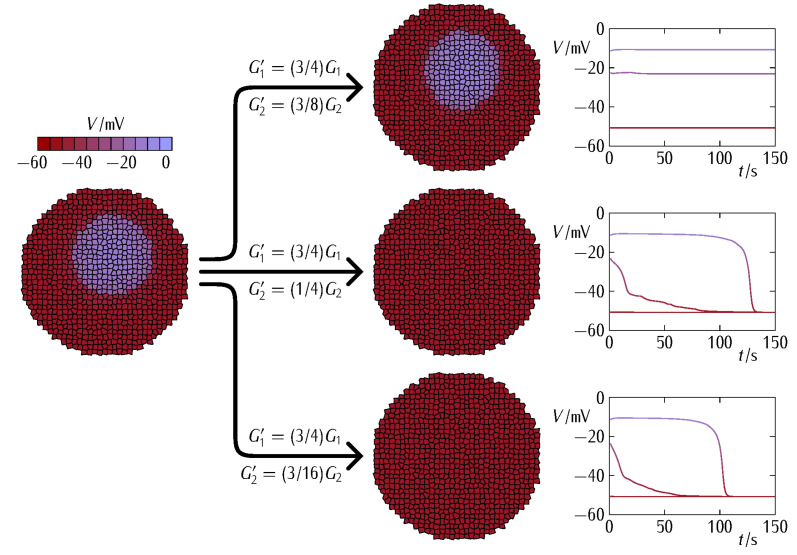
The cell system changes due to a decrease in the conductances $G_{1}$ and $G_{2}$ to the new values $G_{1}^{’}$ (fixed decrease) and $G_{2}^{’}$ (three different decreases) shown in the figure (*left*). The community effect within the depolarized patch can still resist the polarization by the surrounding bulk region if the $G_{2}$ decrease is small (*left*, *top*). Thus, a minimum decrease of $G_{2}$ is needed to weaken the community effect within the patch enough to be polarized (*left*, *intermediate*). Further decreases in $G_{2}$ (*left*, *bottom*) only cause faster patch polarizations, as shown by the electrical potential changes in three cells located at the patch, the surrounding bulk, and the interfacial region (*right*). The single-cell maximum conductances assumed in Equations (1) and (2) are $G_{\mathrm{pol}}^{o}=G_{\mathrm{ref}}^{o}$ and $G_{\mathrm{dep}}^{o}=1.4G_{\mathrm{ref}}^{o}$, with $G_{\mathrm{ref}}^{o}=1_{}^{}\;\mathrm{nS}$. For the case of isolated cells, these conductances give the stable polarized and depolarized potentials $V_{\mathrm{pol}}≃-50_{}^{}\;\mathrm{mV}$ and $V_{\mathrm{dep}}≃-10_{}^{}\;\mathrm{mV}$, respectively [39,47]. The ratio between the initially depolarized patch area and the whole system area is 0.16.

**Figure 5:**
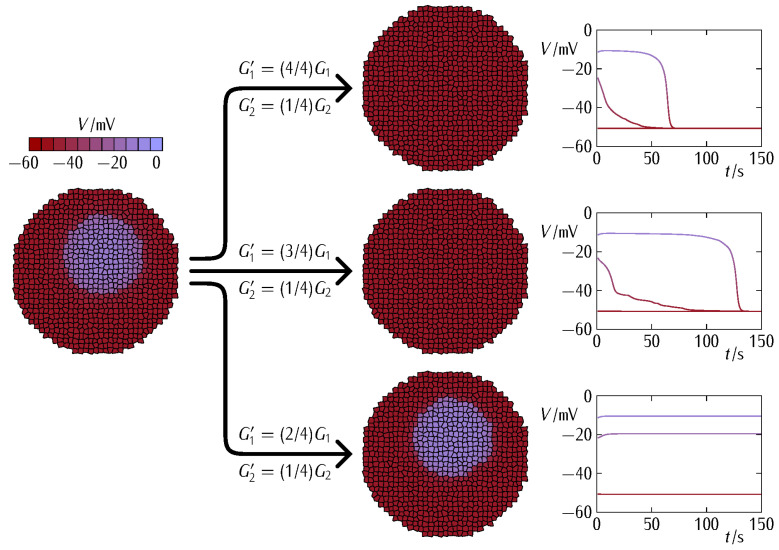
The cell system changes due to a decrease in the conductances $G_{1}$ and $G_{2}$ to the new values $G_{1}^{’}$ (three different decreases) and $G_{2}^{’}$ (fixed decrease) shown in the figure (*left*). At fixed $G_{2}$ decrease, a minimum value of $G_{1}$ is needed for the surrounding bulk to force the polarization of the patch (*left*, *intermediate*). For a lower system connectivity (a lower value of $G_{1}$ ), the patch is effectively isolated from the surrounding bulk region and cannot be polarized (*left*, *bottom*). For a higher value of $G_{1}$ (*left*, *top*) on the contrary, faster patch polarizations are obtained (*right*). The rest of system parameters are those of Figure 4.

In our case, the bioelectrical heterogeneity of Figure 3 (*bottom*) might be due to a distinct complement of ion channel proteins giving different conductances $G_{\mathrm{dep}}^{o}$ and $G_{\mathrm{pol}}^{o}$ as well as to distinct ionic concentrations giving different equilibrium potentials $E_{\mathrm{dep}}$ and $E_{\mathrm{pol}}$ in the two regions [5,39,47]. Note that both cases are fully accounted for in the model Equations (1) and (2). However, we will consider here that all cells have the *same single-cell parameters* and take profit of the polarization state bi-stability [5,39,47] to assume that the individual cells in the two regions of Figure 3 are initially at different polarization states. In this way, we can ascribe the results obtained in Figure 4 and Figure 5 to the changes in the intercellular coupling exclusively because all individual cell parameters remain identical. Figure 4 considers the effect of different $G_{2}$ decreases at fixed $G_{1}$ decrease while Figure 5 considers the effect of different $G_{1}$ decreases at fixed $G_{2}$ decrease. In each case, we show the cell system changes (Figure 4 and Figure 5, *left*) together with the electric potential changes for three cells located at the patch, the surrounding bulk, and the interfacial region (Figure 4 and Figure 5, *right*).

Figure 4 shows that a minimum $G_{2}$ conductance decrease is required to weaken the community effect in the abnormally depolarized central patch just enough to be normalized by the polarized surrounding bulk. Figure 5 shows that a minimum value of $G_{1}$ conductance is needed for the surrounding bulk to force the polarization of the patch. Note that decreasing the values of the conductances $G_{1}$ and $G_{2}$ of Equation (3) impacts on both the patch and the surrounding bulk because of their respective connectivity decreases. These decreases make more difficult not only the resistance of the patch to normalization but also the normalizing effect exerted by the surrounding bulk. 

Taking together, Figure 4 and Figure 5 suggest that different changes in the junction conductances can give non-trivial results that depend not only on the intercellular connectivity but also on the different type of connexins and the particular single-cell bioelectrical states [5] assumed initially. Note also that the above results are difficult to obtain with only one type of connexin gap junction for biologically plausible single-cell and connectivity parameters (not shown here). Indeed, not only the particular values of the conductances $G_{1}$ and $G_{2}$ of Equation (3) but also the different sharpness of the two voltage-gated conductances $G_{ij}$ of Figure 3 (*top*) are crucial here. 

The above effects are modulated further by the asymmetric junction conductance at the interfacial region (Figure 3). Indeed, Equations (3) and (4) show that the intercellular currents at the patch-bulk interfacial region of Figure 3 (*bottom*) that force the patch normalization should depend on the asymmetric type of heterotypic connexin formed. In particular, Figure 3 suggests that the width of the bell-shaped junction conductance $G_{ij}$, which is regulated by the potentials $V_{0,j}$ and $V_{0,i}$, should be important. For instance, no patch regionalization can exist initially if these potentials are high and give thus wide $G_{ij}$ bell functions (the case of Cx43 in Figure 3). These effects are further modulated by the particular values of the conductances $G_{1}$ and $G_{2}$ in Figure 3. Thus, complex cell system dynamics can be obtained even with a relatively simple biophysical model.

Experimentally, effective decreases in the gap junction conductance can be obtained by downregulating the connexin protein expression and by post-translational blocking of the junctions by external agents. In general, low conductances cause restricted intercellular connectivity and can facilitate the spatial regionalization of signaling molecules characteristic of embryogenesis [3,4]. On the contrary, high conductances enhance intercellular interconnectivity and might contribute to the normalization of abnormal cell states in tumorigenesis caused by local changes in the single-cell characteristics [31,39]. However, the fact is that tumorigenic processes are complex and may show apparently contradictory facts regarding the connexin–carcinogenesis association [29,30,32,33,34,35]. In particular, the transference of signaling agents through intercellular junctions may result in different outcomes depending on the target cell state and the molecule to be transferred. We emphasized here a different collective effect: Figure 4 and Figure 5 show that the single-cell responses can also depend on multicellular mean fields regulated by the intercellular coupling conductances [4,36]. 

Additionally, the results suggest the existence of *optimal connectivity values and connexin types for bioelectrical normalization*, although the complex dynamics makes practical application difficult. For example, enhancing the junctional role, e.g., via increased protein expression in the patch, may result in an enhanced *intra-connectivity* and community effect, thus increasing the patch resistance to bioelectrical normalization. Additionally, inhibiting the junctional role at the initial stages of tumorigenesis might decrease the patch-surrounding bulk *inter-connectivity*, making the bioelectrical normalization more difficult and thus giving more opportunities for further growth and dissemination. Therefore, a complex scenario emerges even in the highly simplified case of considering bioelectrical signals only.

**Figure 6:**
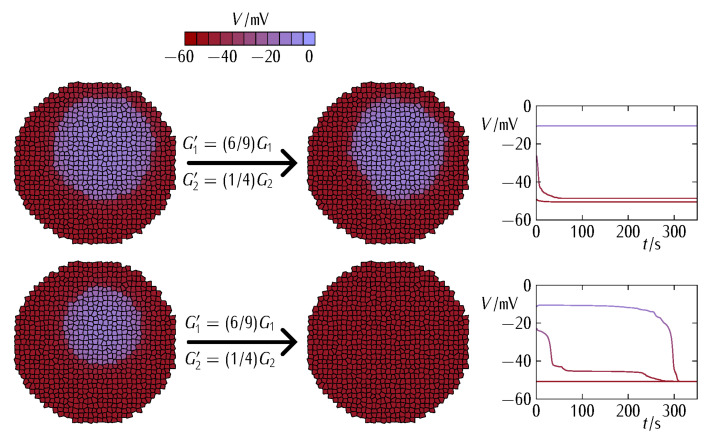
The effect the patch size on the bioelectrical normalization caused by the polarized bulk at constant junction conductance decreases. While the decrease of the intercellular connectivity in the patch allows complete normalization for small sizes (*bottom*), big enough patches can still resist normalization because of the enhanced community effect (*top*). Note that this size effect should also depend on the nature of the heterotypic conductance at the interface between the patch and the surrounding bulk, as suggested by Figure 3. The other system parameters are those of Figure 4.

Figure 6 considers the effect of the patch size on the bioelectrical normalization at constant junction conductance decrease. While the size effect may seem trivial, it can be complicated further by spatial heterogeneities. For instance, no initial electric potential regionalization should be possible for too small patch sizes. In addition, if spatial arrangements of small dispersed patches locally distributed exist in the multicellular aggregate [36], these spots will show low community resistances to polarization, thus enhancing the normalization effect (not shown here). On the contrary, if the depolarized patch of Figure 3 is located in the outer rather than in the inner region of the multicellular aggregate, the normalization effect is significantly diminished, takes more time to be completed and, eventually, cannot occur for big enough patches (not shown here). As to the two-dimensional nature of the cell system used in the simulations, we would expect the mean-field effects reported here to be even stronger for three-dimensional multicellular aggregates because of the increased intercellular connections.

**Figure 7:**
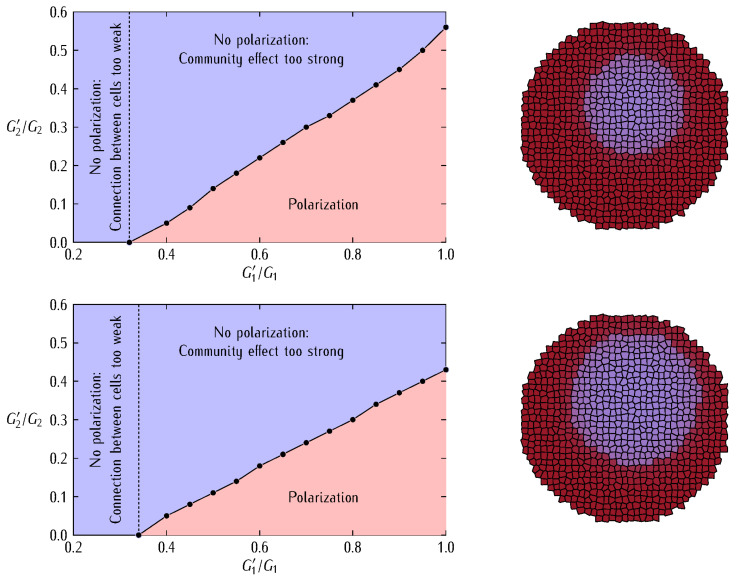
The bioelectrical phase space showing the region where the polarization of the initially depolarized patch is possible (*left*). The *x*-coordinate corresponds to the ratio $G_{1}^{’}/G_{1}$ that parametrizes the assumed decrease in the *minimum* contribution $G_{1}$ to the intercellular connectivity. The *y*-coordinate corresponds to the ratio $G_{2}^{’}/G_{2}$ that parametrizes the assumed decrease in the *maximum* contribution $G_{2}$ of the intercellular connectivity. The initial bioelectrical state of the multicellular aggregate is also shown for two patch sizes (*right*). The patch bioelectrical normalization is not possible for those phase space regions where $G_{1}^{’}/G_{1}$ is *too low* because the weak intercellular connectivity does not allow the forced polarization to proceed in the multicellular aggregate. Additionally, patch normalization is not possible where $G_{2}^{’}/G_{2}$ is *too high* because the strong intercellular connectivity allows the community effect within the patch to resist polarization by the bulk. The single-cell parameters are those of Figure 4 and Figure 5. Note that the phase space regions may also depend on the particular heterotypic conductance at the interface between the patch and the surrounding bulk.

To further understand the relation between the different contributions $G_{1}$ and $G_{2}$ to the total junction conductance (Equation (3) and Figure 3, Figure 4 and Figure 5) and the patch size effect (Figure 6), Figure 7 shows the bioelectrical phase space obtained for the normalization (polarization here) of the depolarized patch. In order to force the bioelectrical normalization of the patch, Figure 7 suggests increasing the minimum conductance term $G_{1}$ and decreasing the maximum conductance term $G_{2}$. In the first case, the increased connectivity within the polarized bulk and at the interfacial region between the bulk and the patch will give intercellular currents high enough to force the normalization (Equation (4)). In the second case, the decreased community effect within the depolarized patch will not allow it to resist the normalization by the polarized bulk. As expected, the above normalizing effects are less effective as the patch size increases. 

**Figure 8:**
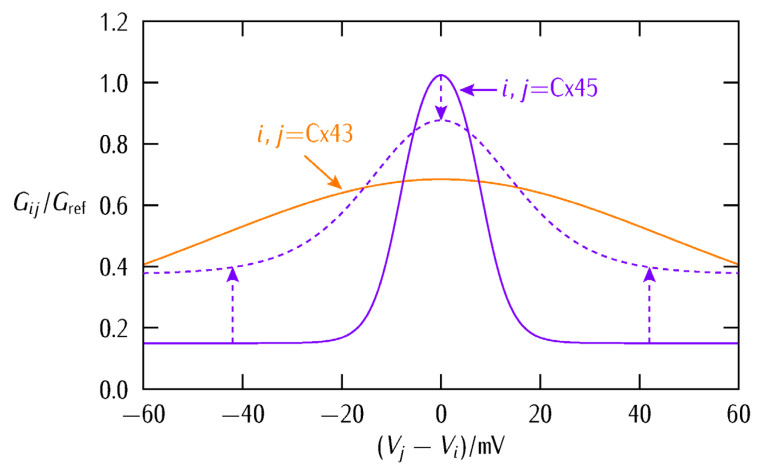
The Cx45 junction voltage-gated conductance tends to the Cx43 one by increasing the minimum conductance term $G_{1}$ and $V_{0,j}=V_{0,j}=V_{0}$ in Equation (3), as shown qualitatively by the arrows and the dashed curve. This transition gives an enhanced connectivity which is almost independent of the intercellular potential difference in the case of the Cx43 junction, contrary to the case of the original sharp Cx45 junction; see Figure 1 here for the experimental conductances together with [21]. This result may suggest *new opportunities for bioelectrical normalization* if the patch junction conductance can be modulated *independently* of the surrounding bulk junction conductance. In particular, the patch conductance should be *increased* at the initial stages of abnormal depolarization to assist the normalization forced by the surrounding bulk while it should be *decreased* at late stages of patch growing and consolidation to avoid the patch resistance to normalization as a whole. Note, however, that this is a purely *bioelectrical* result while connexins and gap junctions have also other *biochemical* and *biomechanical* functions in addition to provide intercellular electrical conductances.

It is important to note that increasing both the minimum conductance term $G_{1}$ and $V_{0,j}=V_{0,j}=V_{0}$ in Equation (3) and Figure 3 makes the voltage-gated Cx45 junction conductance to become close to that of the Cx43 conductance. This result emphasizes further the *enhanced connectivity of the Cx43 junction* compared with that of the Cx45 junction (see Figure 8 and the respective experimental curves of Figure 1). This fact suggests that the Cx43 junction could facilitate the bioelectrical normalization of the small patch by the surrounding bulk at the *initial stages of the abnormal local event*. On the contrary, the Cx43 junction might enhance the influence of a growing abnormal patch on the normal surrounding bulk at *late stages of this event*. Experimentally, the context-dependent role of gap junctions in tumorigenic processes has been noted previously [29,32,33,34,35,48,49].

Note that we concentrated here on the different *spatial distribution* of electrical conductances only, ignoring the fact that distinct connexins might show *different transcriptional and half-life times*. In other words, not only the spatial but also the dynamical changes in the connexin subunit composition of the respective hemichannels can influence the enhancement or shutdown of the interfacial communication. The problem of a time and voltage-dependent protein transcription has also been analyzed by us recently [5,36]. In fact, additional simulations for different single-cell parameters, connexin types (Figure 3, *top*), junction conductance changes, and region characteristics can be conducted and the results of Figure 3, Figure 4, Figure 5, Figure 6, Figure 7 and Figure 8 constitute only representative examples of the possible model outcomes [5,36]. 

We wish to concentrate now on the biophysical insights gained and their connection with real systems. In particular, Figure 3, Figure 4, Figure 5 and Figure 6 suggest that (i) a dynamic voltage-gated intercellular connectivity constitutes a powerful regulatory mechanism to permit or suppress local bioelectrical heterogeneities in a multicellular system and (ii) cells with different connexins can participate in the establishment of instructive bioelectrical patterns that may activate subsequent voltage-dependent phenomena. For instance, changes in the Cx43/Cx45 ratio can influence the transition from quiescence to excitation in myometrium [26] where it has been suggested that as labor approaches, Cx45 is downregulated to permit the spread of electric signals. Note here that the high sharpness of the intercellular conductance corresponding to the homotypic Cx45/Cx45 (Figure 1B) gap junction compared with the Cx43/Cx43 (Figure 1A) case would make an efficient bioelectrical signal propagation in this cell system difficult [26].

The particular connexin composition of the gap junctions influences their function (Figure 3 and Equation (3)). If we consider that the polarized cell state is reminiscent of quiescent cells while the depolarized state is associated with proliferating cells [3,25], the bioelectrical concepts considered here may also be relevant to the tumorigenic role of specific connexin junctions. In this context, previous studies that analyzed the role of Cx46 promoter in lens and other hypoxic tissues concluded that loss of Cx46 upregulates Cx43 in the cell culture, suppressing tumor growth. Other reports have found that Cx43 acts as a positive regulator of stem cell differentiation [48] and thus this connexin is generally considered as anti-tumorigenic and beneficial for survival time [33,34]. Note the wide potential window where Cx43 is fully conductive and allows thus high intercellular coupling compared with other connexins (Figure 1, Figure 3, and Figure 8). Despite this qualitative insight, a limitation of our simulations is that the above connexins may show other non-junctional roles not considered here [49]. Thus, we should look for those cases where bioelectrical effects are clearly involved.

Cancer stem cells (CSCs) express Cx46 while non-CSCs predominantly express Cx43 and during differentiation, Cx46 is reduced while Cx43 is increased [14]. The difference between the Cx43 and Cx46 connexin proteins has been further reflected in distinct intercellular communication and reduced resting membrane potential in the case of CSCs [14]. Note here that Cx46 shows a voltage-gated response which is closer to Cx45 than to Cx43 in Figure 1 [50,51,52]. Thus, our bioelectrical approach suggests that both Cx45 and Cx46 should be more effective than Cx43 in the isolation of the inner patch against the normalization by the surrounding bulk because of their different voltage-gating dependences, as shown by Figure 3 for the case of the Cx45 and Cx43 junctions.

In summary, the pro-tumorigenic or inhibitor roles of particular gap junctions suggests *context-dependent* therapeutic actions based on the transcriptional control of specific connexin levels together with the postranslational modification of the junction function using inhibitors and blockers [14,32,33,34]. These results can also be relevant to the design of synthetic bioelectric systems, whether in vitro [12,53] or in vivo [54]. The problems to be faced in practical applications are the variety of bioelectrical responses that can be obtained as a function of the connexin type, intercellular coupling degree, and patch size, as shown in Figure 3, Figure 4, Figure 5 and Figure 6. This fact should make it difficult to predict the outcomes of using gap junction enhancers and inhibitors [14,32,33,34].

In particular, we cannot give more concrete, specific relations to cancer growth and treatment because of the following facts:

(i) our approach is *bioelectrical* and focused only on the *junctional* effects. Other *biochemical* effects of connexin proteins are omitted here. For instance, these proteins may exchange specific small molecules between cells as well as between cells and the extracellular space. Thus, the outcomes associated with this exchange will depend not only on the conductive state of the junction but also on the particular signaling permeant being transferred between cells. Additionally, connexins can play *non-junctional* roles [49], mediating complex intracellular protein–protein interactions;
(ii) although the loss of the intercellular junction communication could be an *early event* in tumorigenesis, there remains the possibility of gap junction restoration in more advanced tumor stages [35], with partial recovery of the community effect within the patch at *later times.* Thus, enhancing the intercellular gap junction communication may give different outcomes at distinct tumor time stages;
(iii) gap junctions are also involved in *biomechanical* effects such as cell detachment and migration that are not accounted for in our bioelectrical model.

Taken together, the above facts have implications for specific therapies targeting the role of gap junctions and make it difficult to predict the outcomes of using specific gap junction enhancers and inhibitors [14,32,33,34]. With the above caveats in mind, however, we believe that the bioelectrical mean field results discussed here deserve more attention as a complement to traditional biochemical approaches based on molecular single-cell characteristics.

## 4. Summary

We computationally showed that cells may express different connexin proteins as a way to regionalize membrane potentials in multicellular aggregates (Figure 3, Figure 4, Figure 5 and Figure 6) and relate the connexin composition of the intercellular gap junction with different stages of cancer bioelectricity. Additionally, we suggested that enhancing the multicellular connectivity, e.g., via increased connexin expression and gained gap junction function, does not necessarily contribute to bioelectrical normalization (Figure 4). In principle, two normally and abnormally polarized regions which are *highly intra-connected* but *poorly inter-connected* could coexist because of their *high internal* connectivity and *low interfacial* connectivity. Remarkably, this result suggests that from a purely bioelectric viewpoint, the reduction rather than the increase of specific connexin levels may be a suitable therapeutic approach in some cases and time stages.

While cell potentials are not transcription factors themselves, they can influence transcription through biochemical and biomechanical downstream processes [3,5,8,9,55]. Thus, if the cell fate is not terminal and some epigenetic barriers can be manipulated via bioelectrical inputs, cellular reprogramming and large-scale morphogenesis might benefit from the spatio-temporal correlation of cell potentials at the multicellular level, as suggested by Levin and coworkers [3,5,9,15,56]. At this point, it is tempting to speculate that the forced normalization of an abnormally depolarized patch by the polarized surrounding bulk might constitute a control mechanism avoiding local depolarization and subsequent proliferation in a quiescent multicellular aggregate [36,39]. This normalization may fail, however, if the *interfacial connectivity is low*, as it may occur in the initiation of tumorigenic processes [29,39,57]. In addition, it might also fail if the *intercellular connectivity within the abnormal patch is high* and opposes to normalization, as it might occur in more advanced tumor stages. Note, however, that the biological complexity of real systems is not fully captured by our bioelectrically-based approach: modifying the connexin expression or introducing gap junction inhibitors may also cause unintended effects because of the ubiquitous connexin presence and the different functions of these proteins. Additionally, we considered only the junctional role of connexin proteins as transducers of electrical signals, ignoring the transference of biochemical signals. In addition, connexins can show channel-independent functions that are not only stage but also tissue specific, thus displaying both pro- and anti-tumorigenic effects [49,58].

Experimentally, the relevance of bioelectrical activity in cell cycle and proliferation has been documented [58,59,60,61]. In particular, the normally polarized cell state may be reminiscent of quiescent cells while the abnormally depolarized state is associated with proliferating cells [25,59,60]. As to the multicellular states, they emerge as complex outcomes that depend not only on the above single-cell states but also on the intercellular coupling in the patch, the surrounding bulk, and the interfacial region between them [36,39]. Thus, although the single-cell characteristics are crucial, we believe also that the bioelectrical coupling in multicellular aggregates warrants further study. In this scenario, Equations (1)–(4) constitute a minimum model based on conductances ultimately related to specific proteins. In principle, the transcription, translation, and post-translational gating of these ion channel and junction proteins are amenable to external modulation and future therapeutic strategies [3,12,13,15,45,56,62,63,64,65,66,67,68,69,70,71]. Consequently, the bioelectrical patterns and their encoded information could be externally regulated by acting on multicellular mean field phenomena such as electrical potential, potassium, and calcium waves [3,4,5,9,72,73] as a complementary procedure to addressing individual cell characteristics. In this context, the intercellular gap junctions can offer future opportunities [10,28,29,30,31,32,33,34,35,74,75]. Indeed, it has been suggested that ion channel drugs can be used as electroceuticals for treatment of cancer, given appropriate computational modeling tools [76,77], as they have been used for other instances of the control of collective cell behavior in regenerative medicine approaches [78], and repair of birth defects [15,20]. Continued interplay between computational modeling and testing in vivo is sure to reveal fascinating dynamics with practical relevance for biomedical repair and synthetic bioengineering.

## Data Availability

The data presented in this study are available on request from the corresponding author.
